# circUQCRC2 promotes asthma progression in children by activating the VEGFA/NF‐κB pathway by targeting miR‐381‐3p

**DOI:** 10.1002/kjm2.12868

**Published:** 2024-06-20

**Authors:** Li‐Juan Yang, Shu‐Xiang Sui, Qing‐Hua Zheng, Min Wang

**Affiliations:** ^1^ Department of Pediatrics Dongying People's Hospital (Dongying Hospital of Shandong Provincial Hospital Group) Dongying City Shandong Province China

**Keywords:** childhood asthma, circUQCRC2, miR‐381‐3p, VEGFA/NF‐κB

## Abstract

This study targeted to explore circUQCRC2's role and mechanism in childhood asthma. A mouse model of ovalbumin‐induced asthma was established to evaluate the effects of circUQCRC2 on childhood asthma in terms of oxidative stress, inflammation, and collagen deposition. The effects of circUQCRC2 on platelet‐derived growth factor‐BB (PDGF‐BB)‐induced smooth muscle cells (SMCs) were evaluated, the downstream mRNA of miRNA and its associated pathways were predicted and validated, and their effects on asthmatic mice were evaluated. circUQCRC2 levels were upregulated in bronchoalveolar lavage fluid of asthmatic mice and PDGF‐BB‐treated SMCs. Depleting circUQCRC2 alleviated tissue damage in asthmatic mice, improved inflammatory levels and oxidative stress in asthmatic mice and PDGF‐BB‐treated SMC, inhibited malignant proliferation and migration of SMCs, and improved airway remodeling. Mechanistically, circUQCRC2 regulated VEGFA expression through miR‐381‐3p and activated the NF‐κB cascade. circUQCRC2 knockdown inactivated the NF‐κB cascade by modulating the miR‐381‐3p/VEGFA axis. Promoting circUQCRC2 stimulates asthma development by activating the miR‐381‐3p/VEGFA/NF‐κB cascade. Therefore, knocking down circUQCRC2 or overexpressing miR‐381‐3p offers a new approach to treating childhood asthma.

## 1 INTRODUCTION

Asthma is a heterogeneous disease with complex etiology and is a common chronic respiratory disease worldwide. According to the World Health Organization, approximately 334 million people worldwide are affected by asthma.^1^ The early symptoms of asthma in the vast majority of people begin in childhood, especially in early school age. Asthma is closely related to airway inflammation, and childhood asthma is a chronic airway inflammatory response jointly involving a variety of airway cells and mediators, and its main characteristics are wheezing, dyspnea, chest tightness, and cough.^2^, ^3^

Circular RNAs (circRNAs) are a new class of endogenous noncoding RNAs, which form a covalent closed ring by counterbalance splicing and have high tissue specificity and structural stability.^4^ circRNAs regulate gene expression by mediating microRNA (miRNA), interacting with RNA‐binding protein, and interfering with precursor mRNA processing.^5^ There is increasing evidence that circRNAs are crucial regulators of asthma‐related diseases, such as circHIPK3,^6^ circ_406961,^7^ and circZNF652.^8^ circRNA ubiquinol‐cytochrome c reductase core protein 2 (UQCRC2) (circ_0038467) generated by counterbalance splice of UQCRC2 mRNA can protect 16HBE cells from lipopolysaccharide (LPS)‐induced cytotoxicity.^9^ Moreover, circUQCRC2 aggravates inflammatory response and oxidative stress in LPS‐treated human bronchial epithelioid cells.^10^ Nevertheless, circUQCRC2's mechanism in asthma remains unclear.

miRNA is a noncoding, highly conserved RNA molecule composed of about 20 nucleotides, which can regulate gene expression at the translation level.^11^ A large number of miRNAs have been reported to play a role in asthma. For example, miR‐146a and miR‐106b are positively correlated with total immunoglobulin E in children with asthma.^12^ Zhang et al. showed that miR‐410‐3p is involved in airway remodeling in childhood asthma.^13^ Yang et al. proposed that miR‐21 regulates the proliferation of bronchial epithelial cells by activating the transforming growth factor‐β1/Smad signaling pathway, and it is positively correlated with the severity of childhood asthma.^14^ A better understanding of miRNAs may identify targets for new therapies for severe asthma.^15^ One of the genes regulated by miRNA is vascular endothelial growth factor A (VEGFA).^16^ VEGFA is a major regulator of angiogenesis and vascular permeability.^17^ It has been indicated that vascular endothelial growth factor (VEGF) promotes pathological features of asthma, such as mucus secretion, collagen deposition, and smooth muscle hyperplasia.^18^ Its ability to activate signaling pathways in cancer leads to proliferation, differentiation, migration, and invasion of cancer cells. Nuclear factor‐kappa B (NF‐κB) is related to the pathophysiological process of airway diseases by regulating chemokines, cytokines, and adhesion molecules.^19^ Wang et al. suggested that the NF‐κB pathway could induce VEGFA expression in colorectal cancer.^20^ In addition, NF‐κB pathway can promote the expression of VEGFA.^21^ However, it is not clear whether they have a regulatory relationship with children's asthma.

The study explores the interaction and functional correlation between circUQCRC2 and miR‐381‐3p and further reveals the regulatory role of circUQCRC2 in asthma, thus providing a potential pathway for therapeutic intervention of children asthma in children.

## 2 MATERIALS AND METHODS

### 2.1 Animal model

Fifty 2‐week‐old BALB/c female mice were provided by SLAC Laboratory Animal Co., Ltd. (Shanghai, China) and raised following the experimental Animal Management Guidelines. This experimental protocol was approved by the Dongying People's Hospital (Dongying Hospital of Shandong Provincial Hospital Group) Committee. After adaption for 1 week, mice were subcutaneously injected with 20 μg ovalbumin (OVA) (Sigma, MO, USA) emulsified in 2 mg aluminum hydroxide adjuvant at the groin, back, neck, and foot pads on Day 0 and Day 14, respectively. From Day 22 to Day 28, the treated mice were atomized with 1% OVA or equal volume 0.1 M phosphate‐buffered saline (PBS) with a spray atomizer (BARI, Germany) for 30 min, once a day. At 2 h after each atomization, 50 μL shRNA lentiviral vector (sh‐circUQCRC2, CCACTTCACAAGTGCAAGAAT; GenePharma, Shanghai, China) with a concentration of 3 mg/mL was injected into the trachea of mice, and an equal amount of sh‐NC was injected as a negative control. All mice were euthanized on Day 29 by cervical dislocation under pentobarbital sodium (40 mg/kg) anesthesia. Blood and lung samples were taken. The blood samples were centrifuged at 1000 × *g* for 10 min, and the upper serum was collected and stored at −80°C. Part of the lung samples were fixed with 4% paraformaldehyde for hematoxylin and eosin (HE) staining, and part were frozen in liquid nitrogen and stored at −80°C for biochemical analysis.

### 2.2 Bronchoalveolar lavage fluid collection

After blood collection, mouse lung tissue was exposed, and the right lung was bound. A tracheal catheter was inserted into the left lung, lavage with 0.3 mL PBS three times to obtain bronchoalveolar lavage fluid (BALF). BALF was centrifuged at 4°C at 800 × *g* for 10 min, and the supernatant was kept at −80°C. The precipitates were re‐suspended in 0.1 mL PBS, and cell counts were measured with a blood cell counter (Hemavet 950FS; Drew Scientific, CT, USA).

### 2.3 HE staining

The fixed lung tissue was dehydrated with gradient alcohol in turn and impregnated with paraffin at 56°C for at least 30 min. The tissue blocks were made into 5‐μm slices, dewaxed, rehydrated, and stained with HE solution (Sigma‐Aldrich). The slices were observed with an optical microscope (Leica, Germany).

### 2.4 Masson staining

The paraffin‐embedded lung tissue was cut into 5 mm and stained with Weigert solution (Sigma‐Aldrich) for 5–10 min. Next, the slices were treated with ponceau acid solution for 5–10 min, soaked in 2% acetic acid solution for 1 min, and differentiated in 1% phosphomolybdic acid solution for 3–5 min. Subsequently, the slices were treated with aniline blue for 5 min, immersed in 0.2% acetic acid solution for 1 min, infiltrated with xylene, and fixed with neutral glue. Images were captured by Nikon Microscopy (Japan) and the collagen deposition area was calculated using ImageJ software.

### 2.5 Cell culture and transfection

Mouse SMCs (CP‐M158) were purchased from Procell Life Science & Technology Co., Ltd. (Wuhan, China). SMCs were cultured in a 5% CO_2_ incubator at 37°C in a specific complete medium (Procell), which was renewed every 3 days until cell confluence. The cells were passaged in Hanks balanced salt mixture (Gibco, NY, USA) containing 0.25% trypsin and 1 mM ethylenediamine tetraacetic acid and collected at passages 4–6. A cell model of asthma was established by using 25 ng/mL platelet‐derived growth factor‐BB (PDGF‐BB) for 24 h. Small interfering RNA of circUQCRC2 (si‐circUQCRC2, ATCCGCAGACTCAGATTTTAT), negative control (si‐NC), VEGFA overexpression plasmid (pcDNA3.1‐VEGFA), empty plasmid (pcDNA3.1‐NC), miR‐381‐3p mimic, miR‐381‐3p inhibitor, mimic NC, and inhibitor NC were synthesized by GenePharma. At 80% confluence, SMCs were transfected using Lipofectamine™ 2000 (Invitrogen, CA, USA).

### 2.6 Subcellular localization and RNase R digestion

RNA from cytoplasmic and nuclear parts was isolated using the PARIS™ Kit Protein and RNA Separation System Kit (Thermo Fisher Scientific, MA, USA). circUQCRC2 in the nucleus and cytoplasm was determined by real‐time reverse transcriptase polymerase chain reaction (RT‐qPCR).

RNA (5 μg) was extracted from SMCs using TRIzol reagent and mixed with 20 U/μL RNase R (3 U/μg; Epicenter Technologies, WI, USA) at 37°C for 30 min. circUQCRC2 and linear UQCRC2 were measured using RT‐qPCR.

### 2.7 Cell proliferation

Cell Counting Kit‐8 (CCK‐8) kit (Dojindo, Kumamoto, Japan) detected cell proliferation. In short, 2 × 10^3^ cells/well (100 mL) were added to a 96‐well plate. Then, at 0, 24, 48, and 72 h, 10 μL CCK‐8 solution was supplemented to each well and detected at 37°C for 1.5 h. Optical density at 450 nm was measured using a Spectra Max 250 spectrophotometer (Molecular Devices, CA, USA).

### 2.8 Enzyme‐linked immunosorbent assay

Interleukin 6 (IL‐6) (ab222503) and tumor necrosis factor‐α (TNF‐α) (ab208348) in BALF and cell culture supernatant were determined by ELISA kits (Abcam, MA, USA). Superoxide dismutase (SOD) (A001‐3‐2), nitric oxide (NO) (A012‐1‐2), and malondialdehyde (MDA) (A003‐1‐2) in blood and cell supernatant were detected by corresponding commercial kits (Nanjing Jiengcheng Bioengineering Institute, Jiangsu, China).

### 2.9 RT‐qPCR

RNA samples were extracted from lung tissue or SMCs with Trizol reagent (Invitrogen). HiScript II first strand cDNA synthesis kit (Vazyme, Nanjing, China) and miRNA counterbalance transcription kit (TaKaRa) were employed to produce cDNA of mRNA/circRNA or miRNA, respectively. circUQCRC2, UQCRC2, miR‐381‐3p, and VEGFA were analyzed with SYBR Green assay (Cowin Biotech, Beijing, China) with glyceraldehyde‐3‐phosphate dehydrogenase as an internal reference. The multiples were analyzed by $2^{-\Delta \Delta C_{t}}$ method. All primers are shown in Table 1.

**TABLE 1:** Primers.

Genes	Sequences 5′–3′	
circUQCRC2	GCACCCTAACTTCGTCAACC	AGAAATCGACTCTGGCGTCC
UQCRC2	TCAAAGTTGCCCCGAAGGTT	CACCTCCACGGTATTTGGCT
miR‐381‐3p	TACTTAAAGCGAGGTTGCCCTT	GGCAAGCTCTCTGTGAGTA
VEGFA	AGATCTGAGCTTCAACCCCTTG	GCAGAAACATTCGCCAAGCA
GAPDH	TTTGGCATTGTGGAAGGGCT	GGAGTTGCTGTTGAAGTCGC

Abbreviations: circUQCRC2, circular RNA ubiquinol‐cytochrome c reductase core protein 2; GAPDH, glyceraldehyde‐3‐phosphate dehydrogenase; miR‐381‐3p, microRNA‐381‐3p; UQCRC2, ubiquinol‐cytochrome c reductase core protein 2; VEGFA, vascular endothelial growth factor A.

### 2.10 Western blot

Lung tissue and SMCs were separately lysed in radioimmunoprecipitation assay lysis buffer (Beyotime, Shanghai, China), and the extracted proteins were quantified by a bicinchoninic acid kit (Beyotime). The protein was mixed with 2X sample buffer and boiled at 95°C for 5 min, separated by 10% sodium dodecyl sulfate–polyacrylamide gel electrophoresis, and transferred to a nitrocellulose membrane, which was closed with 5% skim milk at room temperature for 1 h and then combined with α‐smooth muscle actin (α‐SMA) (#ab7817; Abcam), E‐cadherin (#ab212059; Abcam), VEGFA (#ab46154; Abcam), p‐NF‐κB p65 (#3039; Cell Signaling Technology, MA, USA), and NF‐κB p65 (#8242; Cell Signaling Technology) overnight at 4°C. GADPH was used as an internal control (#ab9485; Abcam). The membrane was washed three times with Tris–HCL and incubated with secondary goat anti‐mouse (#ab205719; Abcam) or goat anti‐rabbit immunoglobulin G (#ab124055; Abcam) at room temperature for 1 h. After development, the protein bands were analyzed by the image‐PRO Plus Image analysis system.

### 2.11 Transwell assay

The Transwell chamber (Corning, NY, USA) was used to evaluate cell migration. In short, SMCs (5 × 10^4^ cells/well) were re‐suspended overnight in 100 μL serum‐free Dulbecco's modified Eagle's medium (DMEM) and added to the parietal chamber with the basal lateral chamber containing 600 μL 10% fetal bovine serum‐DMEM. After 24 h, SMCs that migrated to the lateral chamber were fixed with 95% methanol and stained with 0.1% crystal violet before microscopic imaging in five random light fields.

### 2.12 Dual‐luciferase reporter gene

The starBase database (http://starbase.sysu.edu.cn/) was employed to predict potential binding sequences of miR‐381‐3p to circUQCRC 2 and VEGFA 3′‐untranslated region (UTR). Mutations were constructed into potential binding sequences using site‐specific mutagenesis kits (Stratagene, CA, USA). A partial fragment of circUQCRC 2 or VEGFA 3′‐UTR, including a wild‐type or mutant binding sequence to miR‐381‐3p, was cloned into a pmirGLO luciferase vector (GenePharma). By using Lipofectamine 2000, miR‐381‐3p mimic or miR‐NC and luciferase reporter were co‐transfected into SMCs for 48 h. Then, luciferase activity was analyzed using the luciferase reporter assay System kit (Promega, WI, USA).

### 2.13 RNA immunoprecipitation

The Magna RIP™ RNA‐binding protein immunoprecipitation kit (Millipore, MA, USA) was utilized for the RNA immunoprecipitation assay. Cell extracts were incubated with agarose beads labeled with antibodies against Argonaute‐2 (#ab186733; Abcam) or immunoglobulin G (#ab6789; Abcam). circUQCRC2, miR‐381‐3p, and VEGFA were measured by RT‐qPCR.

### 2.14 Statistical analysis

Data was analyzed using GraphPad Prism 9.0 (GraphPad, CA, USA). Statistical results are expressed as mean ± standard deviation (SD). Student's *t*‐test and one‐way analysis of variance were used, followed by Tukey's test. *p* < 0.05 was considered statistically significant.

## 3 RESULTS

### 3.1 circUQCRC2 is highly expressed in asthma

**FIGURE 1:**
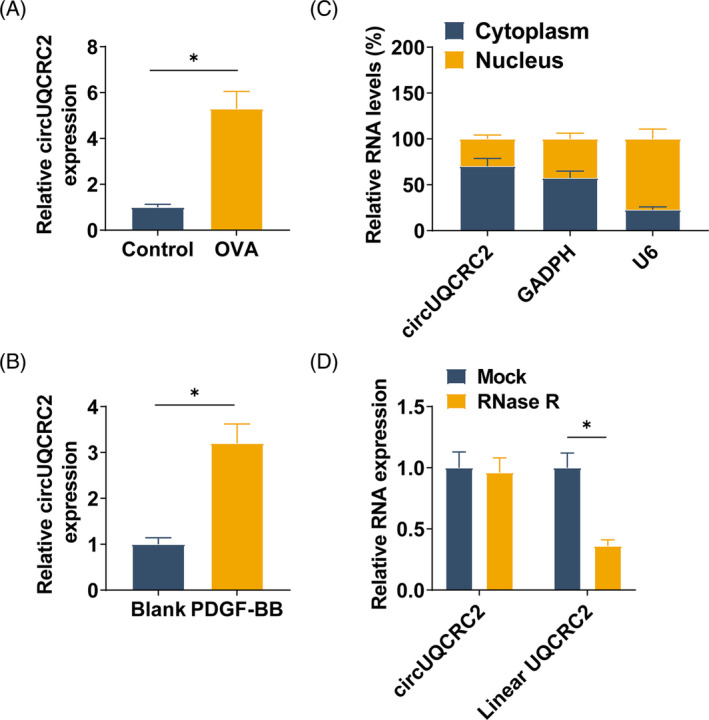
circUQCRC2 is highly expressed in asthma. (A, B) RT‐qPCR detection of circUQCRC2 in OVA‐induced lung tissue and PDGF‐BB‐stimulated SMCs; (C) RT‐qPCR confirmed the subcellular localization of circUQCRC2; (D) after RNase R treatment, RT‐qPCR detected circ‐UQCRC2 and Linear UQCRC2. All experiments were repeated three times, and the data were shown as mean ± SD. *p < 0.05.

After successful modeling of mouse and SMCs, RT‐qPCR detected circUQCRC2 in mouse lung tissue after OVA induction and SMCs after PDGF‐BB stimulation. circUQCRC2 was upregulated in OVA‐treated mouse lung tissue, as well as in SMCs stimulated by PDGF‐BB (Figure 1A,B). circUQCRC2 was mainly distributed in the cytoplasmic portion of SMCs (Figure 1C), suggesting that circUQCRC2 may function at the post‐transcriptional level. RNase R experiment showed that circUQCRC2 was resistant to RNase R, while linear UQCRC2 was reduced after RNase R treatment (Figure 1D), which indicated that circUQCRC2 was a circular transcript.

### 3.2 Knocking down circUQCRC2 improves pathological status in OVA‐induced asthmatic mice

**FIGURE 2:**
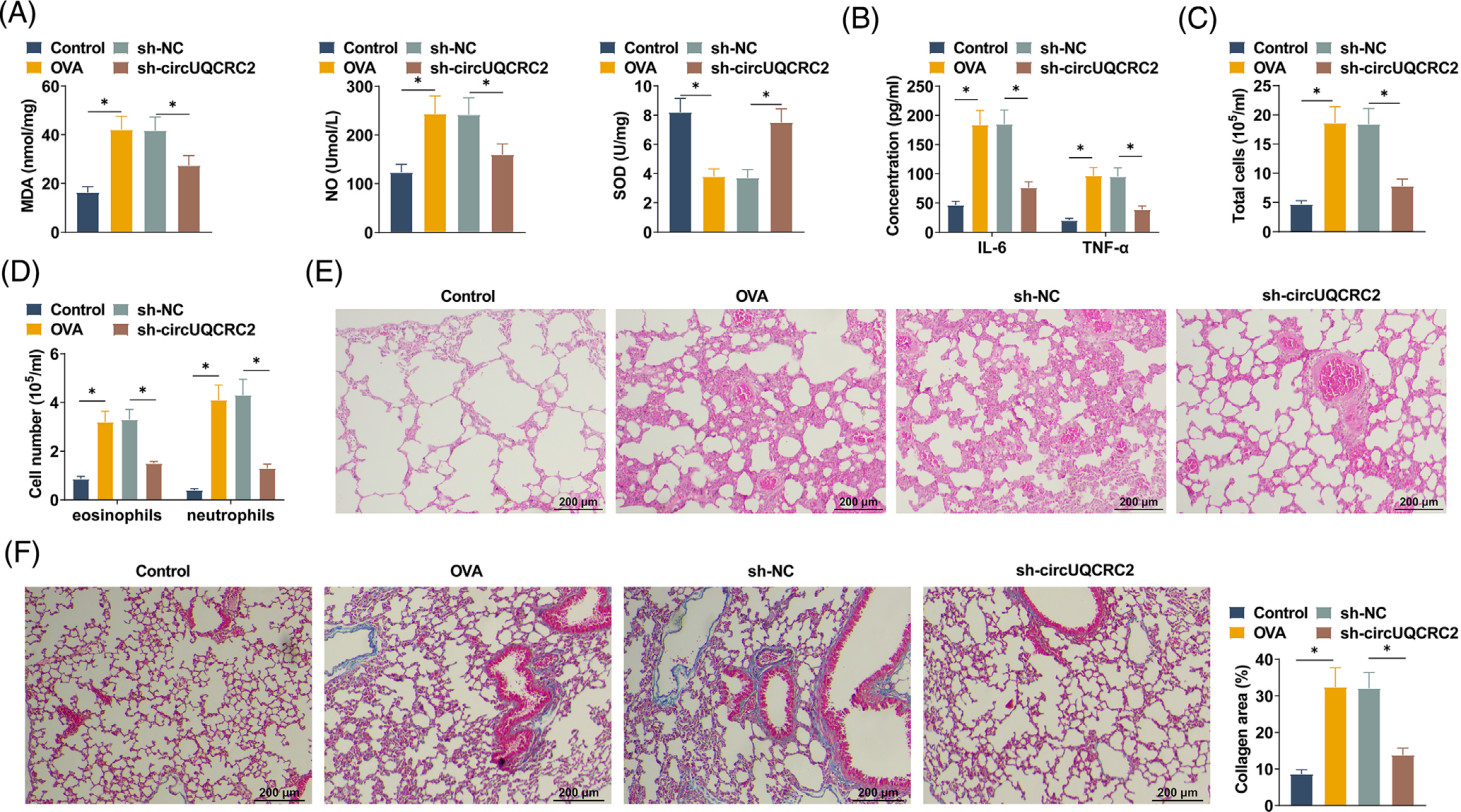
Knocking down circUQCRC2 improves pathological status in OVA‐induced asthmatic mice. (A) ELISA determined MDA, NO, and SOD in blood of mice; (B) ELISA measured the concentrations of IL‐6 and TNF‐α in BALF; (C, D) number of inflammatory cells (eosinophils, macrophages, lymphocytes, and neutrophils) in BALF; (E) HE staining of lung tissues; (F) Masson staining detected collagen deposition in lung tissue. All experiments were repeated three times, and the data were shown as mean ± SD. *p < 0.05.

To investigate the effect of circUQCRC2 on asthma, sh‐circUQCRC2 was administered in asthmatic mice to downregulate circUQCRC2. Then, oxidative stress indices in circulating blood were measured by ELISA kits. MDA and NO contents were increased after OVA treatment, SOD content was decreased, and the above changes were significantly counterbalanced after knocking down circUQCRC2 (Figure 2A). At the same time, IL‐6 and TNF‐α levels were measured in BALF, and OVA‐attacked mice showed elevated levels of IL‐6 and TNF‐α relative to normal mice and then decreased levels after knocking down circUQCRC2 (Figure 2B). The effect of circUQCRC2 knockdown on inflammatory cells in an OVA‐induced mouse asthma model was studied by cell counting. OVA‐induced mice showed a significant increase in total cell inflow and number of cells (eosinophils and neutrophils) compared to normal mice. However, after knocking down circUQCRC2, a significant decrease in cell influx was observed (Figure 2C). Consistent with the above results, lowering circUQCRC2 resulted in a significant reduction in inflammatory cells (Figure 2D). To more visually observe lung injury in mice, HE staining was performed (Figure 2E). OVA‐induced inflammatory cells were infiltrated and accumulated in mouse lung tissue, and the lung structure was obviously deformed. After lowering circUQCRC2, all pathological changes were reduced, and inflammatory infiltration was also attenuated. Masson staining detected collagen deposition in tissues (Figure 2F). OVA‐induced mice showed more collagen deposition and fibrosis in lung tissue, and knocking down circUQCRC2 mitigated asthma‐induced collagen deposition.

### 3.3 circUQCRC2 knockdown inhibits SMC proliferation and migration and improves inflammation and oxidative stress

**FIGURE 3:**
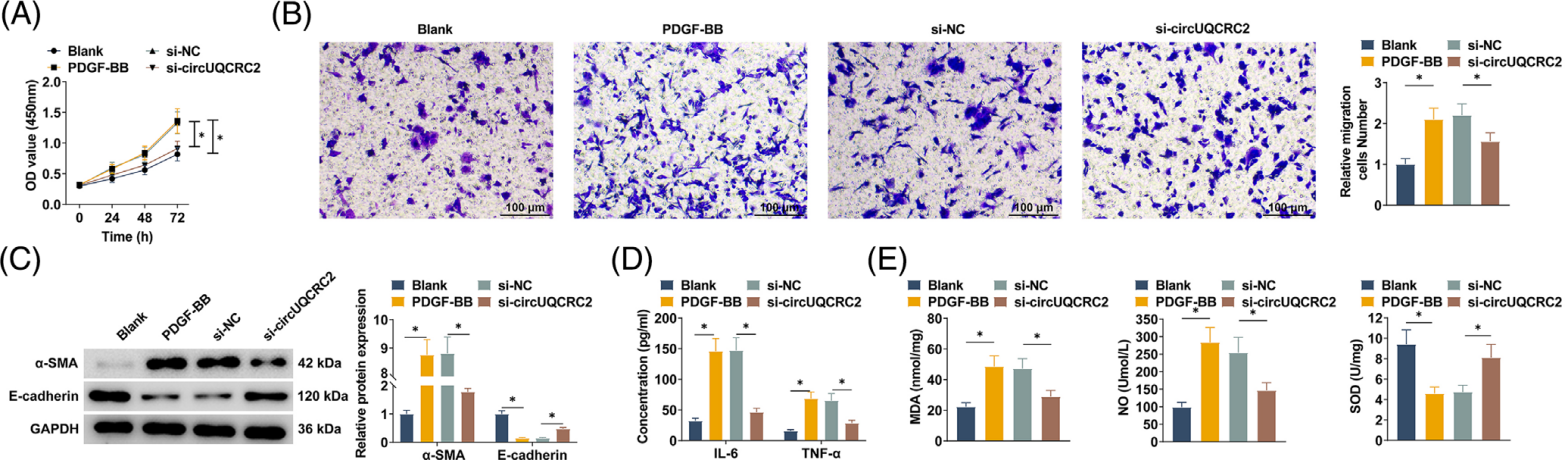
circUQCRC2 knockdown inhibits SMC proliferation and migration and improves inflammation and oxidative stress. PDGF‐BB‐treated SMCs were transfected with si‐circUQCRC2 and si‐NC, respectively. (A) CCK‐8 detected SMC proliferation; (B) Transwell method measured SMC migration; (C) Western blot detected α‐SMA and E‐cadherin in SMCs; (D) ELISA determined the concentrations of IL‐6 and TNF‐α in SMC supernatant; (E) ELISA determined MDA, NO, and SOD levels in SMC supernatant. All experiments were repeated three times, and the data were shown as mean ± SD. *p < 0.05.

To investigate the effect of circUQCRC2 on PDGF‐BB‐induced SMC asthma model in vitro, SMCs were transfected with si‐circUQCRC2 to knock down circUQCRC2. The effect of circUQCRC2 on SMC proliferation and migration was studied by CCK‐8 and Transwell. PDGF‐BB significantly promoted the abnormal proliferation of SMCs, and this abnormal proliferation was inhibited after knocking down circUQCRC2 (Figure 3A). The migration ability of SMCs treated with PDGF‐BB was enhanced, and the migration level of SMC was reduced by knocking down circUQCRC2 (Figure 3B). By measuring α‐SMA and E‐cadherin by Western blot, it could be observed that α‐SMA was increased in PDGF‐BB‐treated SMCs, while E‐cadherin was decreased. Knocking down circUQCRC reduced α‐SMA expression and upregulated E‐cadherin expression (Figure 3C). IL‐6 and TNF‐α in the cell supernatant were detected by ELISA kits. When stimulated by PDGF‐BB, SMC released increased levels of IL‐6 and TNF‐α, while knocking down circUQCRC2 improved the above stimulation (Figure 3D). MDA and NO in SMC after PDGF‐BB stimulation were forced, SOD content was suppressed, and the above changes were counterbalanced when circUQCRC2 was knocked down (Figure 3E).

### 3.4 circUQCRC2 acts as a molecular sponge targeting miR‐381‐3p

**FIGURE 4:**
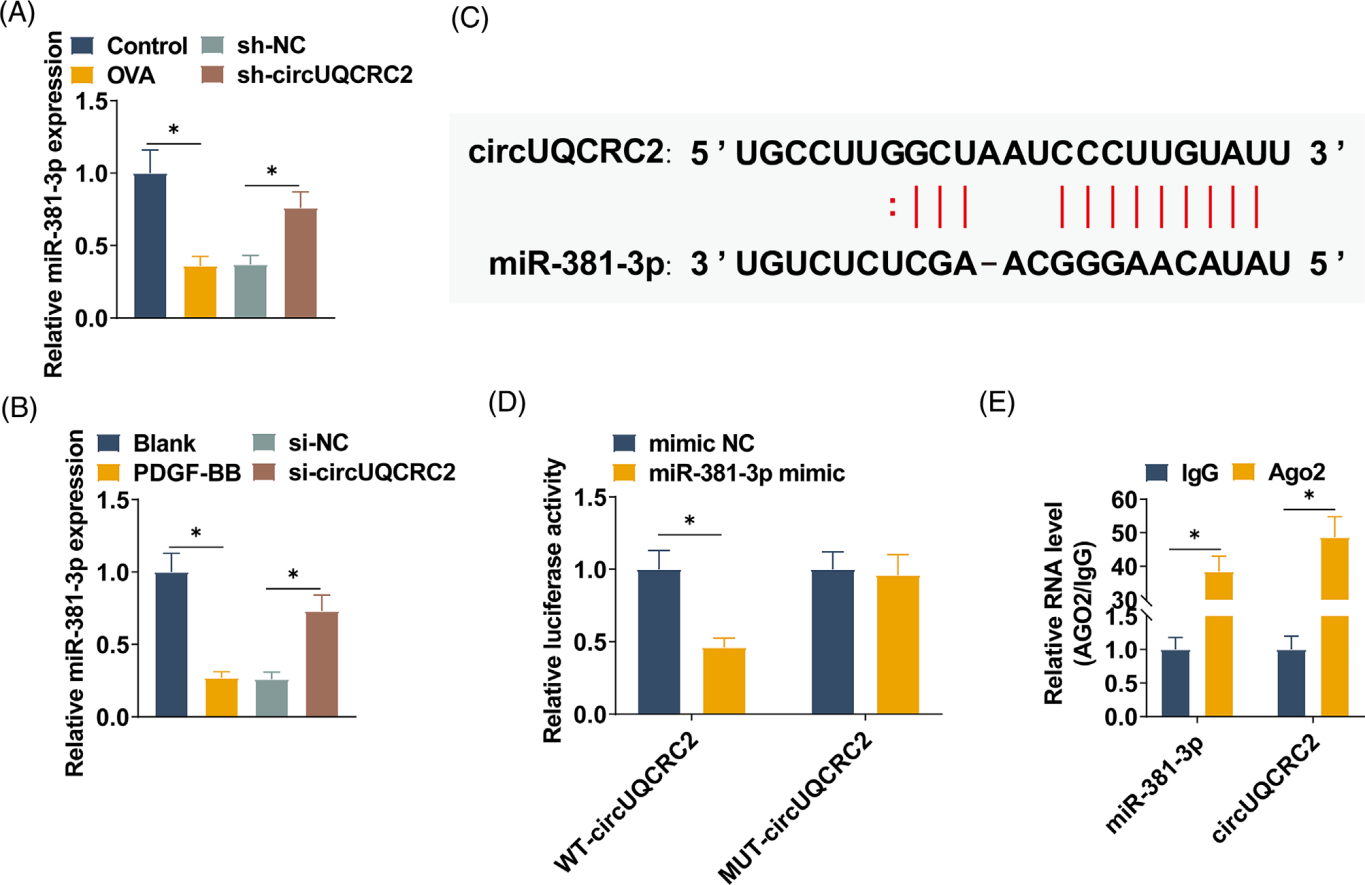
circUQCRC2 is a molecular sponge of miR‐381‐3p. (A, B) RT‐qPCR detection of miR‐381‐3p in mouse lung tissue and SMCs; (C) starBase predicted the binding sites of miR‐381‐3p and circUQCRC2; (D, E) dual‐luciferase reporter gene analysis and RIP analysis verified the targeting relationship between circUQCRC2 and miR‐381‐3p. All experiments were repeated three times, and the data were shown as mean ± SD. *p < 0.05.

RT‐qPCR detected low expression of miR‐381‐3p in lung tissues of asthmatic mice and also in SMCs treated with PDGF‐BB. Depleting circUQCRC2 increased miR‐381‐3p levels (Figure 4A,B). This indicates a negative regulatory relationship between circUQCRC2 and miR‐381‐3p. miR‐381‐3p was predicted to be one of the candidate targets for circUQCRC2 by StarBase software, and the predicted binding sites between circUQCRC2 and miR‐381‐3p were shown in Figure 4C. Moreover, the results of the dual‐luciferase reporter gene showed (Figure 4D) that co‐transfection of miR‐381‐3p mimic and WT‐circUQCRC2 could reduce the luciferase activity of cells. It indicates that miR‐381‐3p interacts with circUQCRC2. RIP results showed (Figure 4E) that miR‐381‐3p and circUQCRC2 were simultaneously enriched in the Ago2 antibody, indicating a spatial interaction between miR‐7‐5p and circUQCRC2 in the RNA‐induced silencing complex.

### 3.5 Depleting miR‐381‐3p counterbalances the therapeutic effect of depleting circUQCRC2 on asthma

**FIGURE 5:**
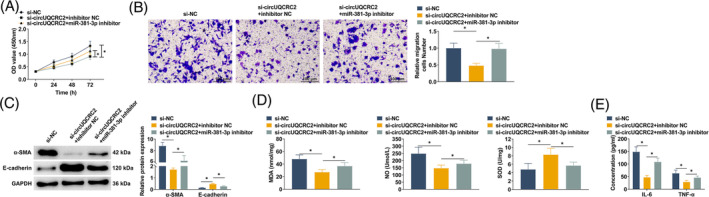
Knocking down miR‐381‐3p counterbalances the therapeutic effect of knocking down circUQCRC2 in asthma. SMCs were transfected with si‐circUQCRC2 and miR‐381‐3p inhibitor. (A) CCK‐8 detected SMC proliferation; (B) Transwell method measured SMC migration; (C) Western blot detected α‐SMA and E‐cadherin in SMCs; (D) ELISA determined MDA, NO, and SOD levels in SMC supernatant; (E) ELISA determined the concentrations of IL‐6 and TNF‐α in SMC supernatant. All experiments were repeated three times, and the data were shown as mean ± SD. *p < 0.05.

SMCs were transfected with si‐circUQCRC2 + inhibitor NC, si‐circUQCRC2 + miR‐381‐3p inhibitor, and si‐NC inhibitor, respectively. Rescue experiments showed that, compared with si‐circUQCRC2 alone, co‐transfection of si‐circUQCRC2 and miR‐381‐3p inhibitor could significantly promote abnormal proliferation of SMCs and counterbalance the inhibitory effect of si‐circUQCRC2 on it (Figure 5A). miR‐381‐3p inhibitor counterbalanced si‐circUQCRC2‐mediated migration inhibition (Figure 5B). α‐SMA protein expression was increased, and E‐cadherin protein expression was downregulated after co‐transfection of si‐circUQCRC2 and miR‐381‐3p inhibitor (Figure 5C). MDA, NO, IL‐6, and TNF‐α were upregulated, and SOD was downregulated in SMCs co‐transfected with si‐circUQCRC2 and miR‐381‐3p inhibitor (Figure 5D,E).

### 3.6 miR‐381‐3p targets VEGFA

**FIGURE 6:**
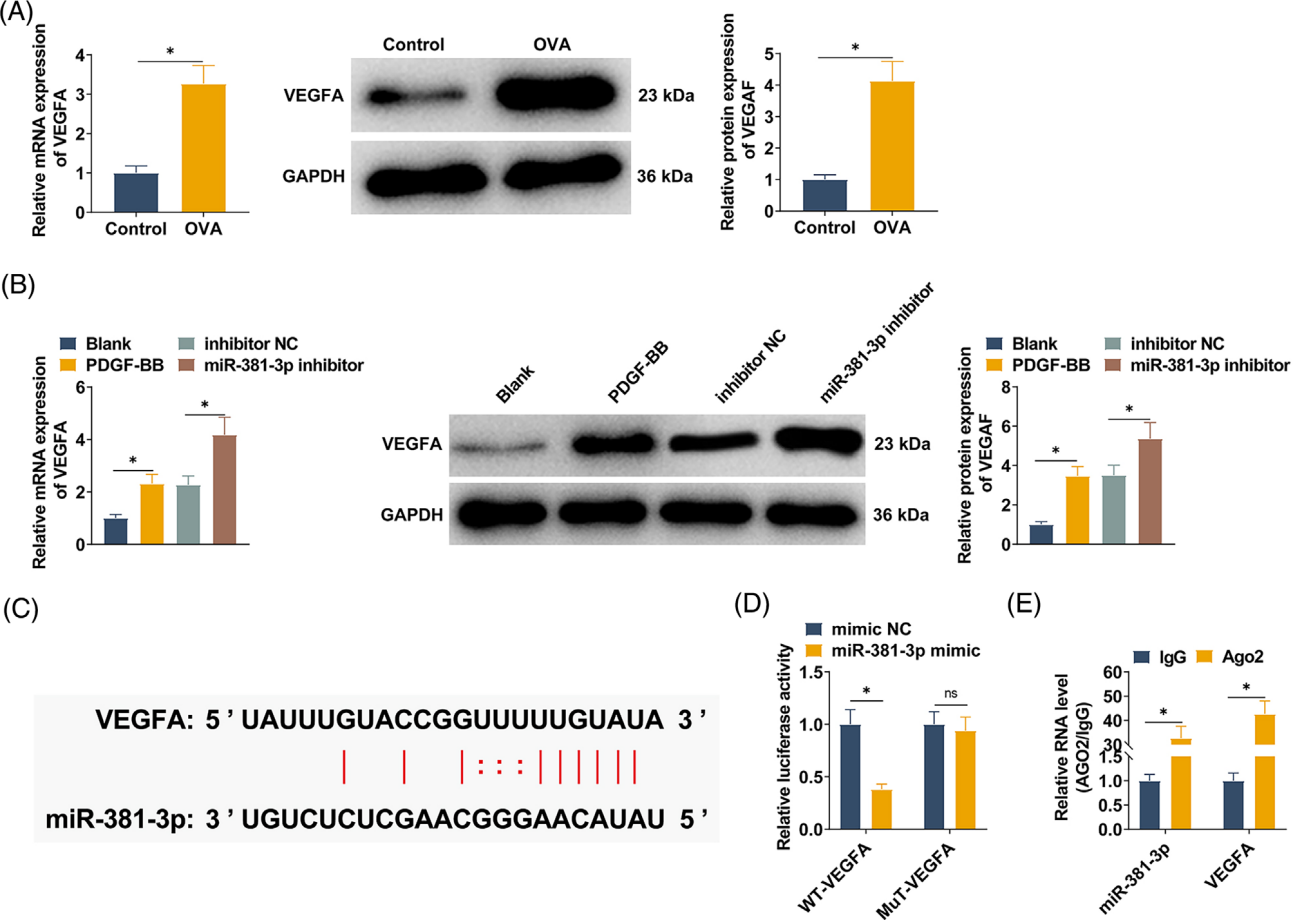
miR‐381‐3p targets VEGFA and activates the NF‐κB cascade. (A, B) RT‐qPCR and Western blot analyzed VEGFA in mice and SMCs; (C) starBase predicted the binding sites of miR‐381‐3p and VEGFA; (D, E) dual‐luciferase reporter gene analysis and RIP analysis verified the targeting relationship between miR‐381‐3p and VEGFA. All experiments were repeated three times, and the data were shown as mean ± SD. *p < 0.05.

RT‐qPCR and Western blot detected the high expression of VEGFA in OVA‐induced mice (Figure 6A). VEGFA was also highly expressed in PDGF‐BB‐treated SMCs, and VEGFA expression was further induced after transfection with miR‐381‐3p inhibitor (Figure 6B). Figure 6C predicted the binding sites of VEGFA and miR‐381‐3p through the StarBase database. Dual‐luciferase reporter gene results showed that miR‐381‐3p mimic co‐transfected with WT‐VEGFA could reduce the luciferase activity, while co‐transfection with MUT‐VEGFA showed no activity against luciferase (Figure 6D). In the experimental results of RIP, miR‐381‐3p and VEGFA were enriched when the Ago2 antibody was used (Figure 6E), which further demonstrated the interaction between miR‐381‐3p and VEGFA.

### 3.7 VEGFA upregulation counterbalances the therapeutic effect of knocking down circUQCRC2

**FIGURE 7:**
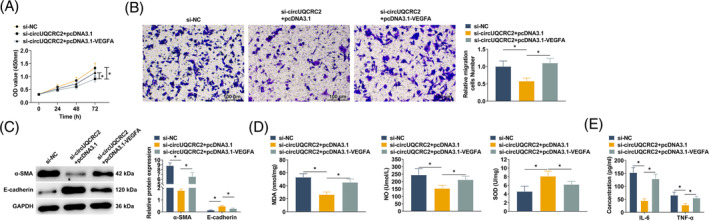
Overexpressing VEGFA counterbalances the effect of knocking down circUQCRC2. SMCs were transfected with si‐circUQCRC2 and pcDNA‐VEGFA. (A) CCK‐8 detected SMC proliferation; (B) Transwell method measured SMC migration; (C) Western blot detected α‐SMA and E‐cadherin in SMCs; (D) ELISA determined MDA, NO, and SOD levels in SMC supernatant; (E) ELISA determined the concentrations of IL‐6 and TNF‐α in SMC supernatant. All experiments were repeated three times, and the data were shown as mean ± SD. *p < 0.05.

To further verify that circUQCRC2 influences VEGFA's biological function in childhood asthma by regulating miR‐381‐3p, SMCs were transfected with si‐NC, si‐circUQCRC2, si‐circUQCRC2 + VEGFA overexpressing plasmid. Knocking down circUQCRC2 significantly inhibited the proliferation and migration of SMCs (Figure 7A,B). Moreover, knocking down circUQCRC2 downregulated α‐SMA protein and increased E‐cadherin protein (Figure 7C). Knocking down circUQCRC2 could suppress MDA and NO and increase SOD (Figure 7D). Furthermore, knocking down circUQCRC2 improved inflammation and inhibited IL‐6 and TNF‐α levels (Figure 7E). However, VEGFA upregulation partially counteracted these effects (Figure 7A–E).

### 3.8 circUQCRC2 activates the VEGFA/NF‐κB cascade by targeting miR‐381‐3p

**FIGURE 8:**
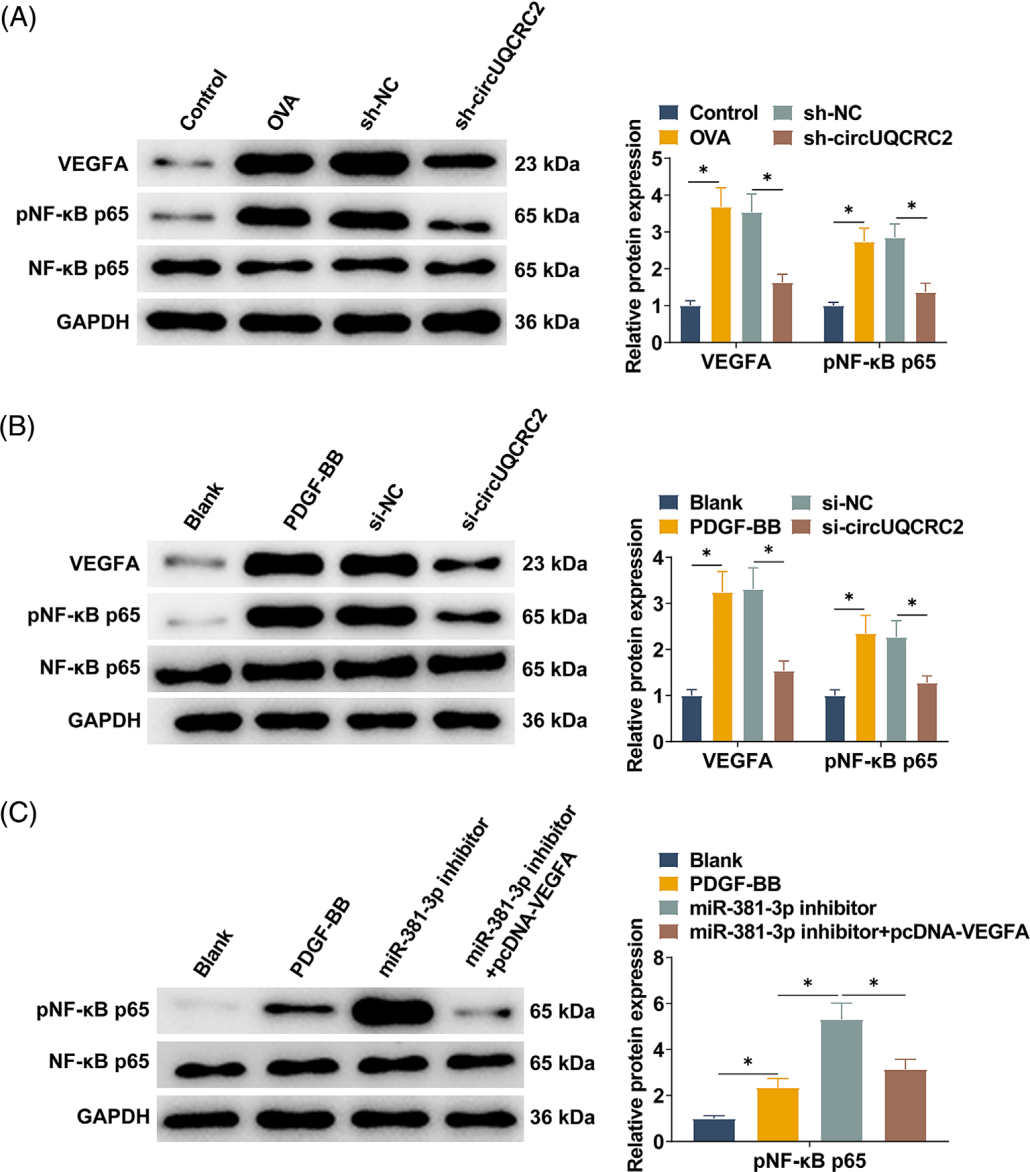
circUQCRC2 activates the VEGFA/NF‐κB cascade by targeting miR‐381‐3p. (A) Western blot analyzed VEGFA and phosphorylated NF‐κB in mouse lung tissue; (B) Western blot analyzed VEGFA and phosphorylated NF‐κB in SMCs; (C) Western blot investigated the relationship between miR‐381‐3p, VEGFA, and pathway protein NF‐κB. All experiments were repeated 3 times, and the data were shown as mean ± SD. *p < 0.05.

VEGFA is thought to promote NF‐κB phosphorylation that leads to inflammation. NF‐κB activation and VEGFA expression in lung tissue were detected by Western blot. High expression of VEGFA in lung tissue of asthmatic mice was accompanied by a significant increase in the phosphorylation of NF‐κB. After circUQCRC2 was knocked down, VEGFA expression in mouse lung tissue was downregulated, and NF‐κB phosphorylation was also reduced (Figure 8A). Western blot technology was further used to explore the regulatory mechanism of the VEGFA/NF‐κB cascade in SMCs. VEGFA expression was increased in PDGF‐BB‐induced SMCs, which activated the NF‐κB pathway and increased NF‐κB phosphorylation. The same results were observed when circUQCRC2 was reduced in SMCs, with decreased VEGFA expression and inhibited NF‐κB phosphorylation (Figure 8B). Western blot technology further investigated the relationship between miR‐381‐3p, VEGFA, and pathway protein NF‐κB. The results showed that PDGF‐BB induced activation of the NF‐κB pathway and increased NF‐κB phosphorylation in SMCs. miR‐381‐3p inhibitor led to a further increase in phosphorylated NF‐κB, which was inhibited after transfection with VEGFA overexpressing plasmid (Figure 8C).

## 4 DISCUSSION

Asthma is the most common chronic nonspecific airway inflammatory disease in childhood, characterized by chronic airway inflammation, airway hyperresponsiveness, and irreversible airway remodeling. Childhood asthma is multifactorial and is closely related to airway inflammation.^22^ In this study, the OVA‐induced asthma mouse model and SMC stimulated by PDGF‐BB were established. circUQCRC2 was upregulated in asthmatic mice and the SMC asthma model. Preliminary evidence suggests a facilitating role for circUQCRC2 in the pathogenesis of asthma in the young. It has been demonstrated that circUQCRC2 plays the same pathogenesis‐promoting role in infantile pneumonia.^10^, ^23^ The pathogenesis of asthma is closely related to oxidative stress and airway inflammation, and air‐to‐air inflammation is closely related to the increase of eosinophils or neutrophils.^24^, ^25^ Eosinophilic airway inflammation is a hallmark of allergic asthma, and oxidative stress processes play an important role in the pathogenesis and progression of asthma.^26^ Levels of antioxidants and oxidative stress markers have also been reported to be associated with asthma severity.^27^ All these studies are in agreement with our results. In the present study, knocking down circUQCRC2 decreased MDA and NO content in BALF and SMCs, increased SOD activity, decreased IL‐6 and TNF‐α expression, decreased eosinophilic and neutrophil counts in BLAF, and improved inflammatory cell infiltration and collagen deposition. Airway remodeling is an important feature of asthma development,^28^ and airway remodeling may occur in the early stages of asthma and is associated with epithelial–mesenchymal transition (EMT) dysfunction.^29^ The abnormal proliferation and migration of airway smooth muscle cells are related to asthma. E‐cadherin^30^ and α‐SMA, two contractile phenotypic marker proteins, are involved in airway remodeling in asthma. Downregulation of E‐cadherin, an important intercellular adhesion protein, is a hallmark of EMT and is accompanied by upregulation of mesenchymal markers, including α‐SMA.^31^, ^32^ In this study, it was observed that knocking down circUQCRC2 prevented abnormal proliferation and migration of SMCs and increased α‐SMA and E‐cadherin levels. It is thus clear that circUQCRC2 is closely associated with cellular inflammation, oxidative stress, and airway remodeling in children with asthma.

The ceRNA hypothesis provides a new mechanism by which noncoding RNAs are involved in regulating biological processes. circRNA can act as a molecular sponge to indirectly regulate gene expression by inducing miRNA.^33^ In this study, circUQCRC2 was mainly distributed in the cytoplasm of SMCs, so it may have the function of ceRNA. The specific binding of miR‐381‐3p to circUQCRC2 was verified. Moreover, inhibiting miR‐381‐3p expression was sufficient to counterbalance the therapeutic effect of circUQCRC2 knockdown on asthma. Moreover, miR‐381‐3p directly targets VEGFA, and an asthma model led to increased VEGFA expression. Xu et al.^34^ analyzed the proinflammatory transcription factor NF‐κB mediating PM2.5 production of VEGFA. Overexpressing VEGFA is believed to effectively regulate airway hyperactivity, chronic airway inflammation, and vascular remodeling.^35^, ^36^ In asthma, the activation of NF‐κB occurs through promoting inflammatory regulators. NF‐κB signaling is influenced by the imbalance between antioxidants and oxidants in airway diseases.^37^ This is consistent with our results that increased VEGFA expression, and increased NF‐κB nuclear translocation in young mouse models of asthma aggravated inflammatory cell infiltration and collagen deposition. Activation of the VEGFA/NF‐κB pathway was associated with increased inflammatory responses in asthmatic mice, manifested in part by promoting IL‐6 and TNF‐α levels. Activation of the VEGFA/NF‐κB pathway also promoted oxidative stress in mouse models of asthma. In addition, overexpressing VEGFA counterbalanced the therapeutic effect of circUQCRC2 knockdown on asthma.

In summary, our finding that circUQCRC2 is one of the mechanisms to regulate children asthma by regulating the miR‐381‐3p/VEGFA/NF‐κB signaling pathway, and other potential mechanisms should be explored by more studies. Therefore circUQCRC2, miR‐381‐3p and VEGFA/NF‐κB may be targets for the treatment of infantile asthma.

## CONFLICT OF INTEREST STATEMENT

The authors declare no conflict of interest.

## References

[1] Papi A , Brightling C , Pedersen SE , Reddel HK . Asthma. Lancet. 2018;391(10122):783–800. 29273246 10.1016/S0140-6736(17)33311-1

[2] Zhai C , Wang D . Baicalin regulates the development of pediatric asthma via upregulating microRNA‐103 and mediating the TLR4/NF‐κB pathway. J Recept Signal Transduct Res. 2022;42(3):230–240. 33730981 10.1080/10799893.2021.1900865

[3] Finkas LK , Martin R . Role of small airways in asthma. Immunol Allergy Clin North Am. 2016;36(3):473–482. 27401620 10.1016/j.iac.2016.03.009

[4] Li X , Yang L , Chen LL . The biogenesis, functions, and challenges of circular RNAs. Mol Cell. 2018;71(3):428–442. 30057200 10.1016/j.molcel.2018.06.034

[5] Salzman J . Circular RNA expression: its potential regulation and function. Trends Genet. 2016;32(5):309–316. 27050930 10.1016/j.tig.2016.03.002 PMC4948998

[6] Lin J , Feng X , Zhang J . Circular RNA circHIPK3 modulates the proliferation of airway smooth muscle cells by miR‐326/STIM1 axis. Life Sci. 2020;255:117835. 32450169 10.1016/j.lfs.2020.117835

[7] Jia Y , Li X , Nan A , Zhang N , Chen L , Zhou H , et al. Circular RNA 406961 interacts with ILF2 to regulate PM_2.5_‐induced inflammatory responses in human bronchial epithelial cells via activation of STAT3/JNK pathways. Environ Int. 2020;141:105755. 32388272 10.1016/j.envint.2020.105755

[8] Wang X , Xu C , Cai Y , Zou X , Chao Y , Yan Z , et al. CircZNF652 promotes the goblet cell metaplasia by targeting the miR‐452‐5p/JAK2 signaling pathway in allergic airway epithelia. J Allergy Clin Immunol. 2022;150(1):192–203. 35120971 10.1016/j.jaci.2021.10.041

[9] Liu G , Wan Q , Li J , Hu X , Gu X , Xu S . Circ_0038467 regulates lipopolysaccharide‐induced inflammatory injury in human bronchial epithelial cells through sponging miR‐338‐3p. Thorac Cancer. 2020;11(5):1297–1308. 32181994 10.1111/1759-7714.13397 PMC7180556

[10] Zhang X , Chen C , Li B , Lu W . Circ‐UQCRC2 aggravates lipopolysaccharide‐induced injury in human bronchial epithelioid cells via targeting miR‐495‐3p/MYD88‐mediated inflammatory response and oxidative stress. Autoimmunity. 2021;54(8):483–492. 34499003 10.1080/08916934.2021.1975273

[11] Gomez JL . Epigenetics in asthma. Curr Allergy Asthma Rep. 2019;19(12):56. 31776749 10.1007/s11882-019-0886-y PMC6986424

[12] Elnady HG , Sherif LS , Kholoussi NM , Ali Azzam M , Foda AR , Helwa I , et al. Aberrant expression of immune‐related microRNAs in pediatric patients with asthma. Int J Mol Cell Med. 2020;9(4):246–255. 33688482 10.22088/IJMCM.BUMS.9.4.246 PMC7936071

[13] Zhang T , Huang H , Liang L , Lu H , Liang D . Long non‐coding RNA (LncRNA) non‐coding RNA activated by DNA damage (NORAD) knockdown alleviates airway remodeling in asthma via regulating miR‐410‐3p/RCC2 and inhibiting Wnt/β‐catenin pathway. Heliyon. 2024;10(1):e23860. 38261955 10.1016/j.heliyon.2023.e23860 PMC10796956

[14] Kang Y , Bai M , Deng L , Fan L , Wang X . MiRNA‐21 regulates bronchial epithelial cell proliferation by activating Tgfβ1/Smad signaling pathway and its correlation with asthma severity in children. Iran J Public Health. 2021;50(10):1973–1982. 35223564 10.18502/ijph.v50i10.7497 PMC8819233

[15] Maneechotesuwan K . Role of microRNA in severe asthma. Respir Investig. 2019;57(1):9–19. 10.1016/j.resinv.2018.10.005 30455067

[16] Cuzziol CI , Castanhole‐Nunes MMU , Pavarino ÉC , Goloni‐Bertollo EM . MicroRNAs as regulators of *VEGFA* and *NFE2L2* in cancer. Gene. 2020;759:144994. 32721475 10.1016/j.gene.2020.144994

[17] Yancopoulos GD , Davis S , Gale NW , Rudge JS , Wiegand SJ , Holash J . Vascular‐specific growth factors and blood vessel formation. Nature. 2000;407(6801):242–248. 11001067 10.1038/35025215

[18] Lee KS , Kim SR , Park HS , Jin GY , Lee YC . Cysteinyl leukotriene receptor antagonist regulates vascular permeability by reducing vascular endothelial growth factor expression. J Allergy Clin Immunol. 2004;114(5):1093–1099. 15536415 10.1016/j.jaci.2004.07.039

[19] Dai R , Niu M , Wang N , Wang Y . Syringin alleviates ovalbumin‐induced lung inflammation in BALB/c mice asthma model via NF‐κB signaling pathway. Environ Toxicol. 2021;36(3):433–444. 33146439 10.1002/tox.23049

[20] Wang R , Ma Y , Zhan S , Zhang G , Cao L , Zhang X , et al. B7‐H3 promotes colorectal cancer angiogenesis through activating the NF‐κB pathway to induce VEGFA expression. Cell Death Dis. 2020;11(1):55. 31974361 10.1038/s41419-020-2252-3 PMC6978425

[21] Liang S , Chen Z , Jiang G , Zhou Y , Liu Q , Su Q , et al. Activation of GPER suppresses migration and angiogenesis of triple negative breast cancer via inhibition of NF‐κB/IL‐6 signals. Cancer Lett. 2017;386:12–23. 27836733 10.1016/j.canlet.2016.11.003

[22] Noutsios GT , Floros J . Childhood asthma: causes, risks, and protective factors; a role of innate immunity. Swiss Med Wkly. 2014;144:w14036. 25539126 10.4414/smw.2014.14036

[23] Zhou G , Duan Y , Lu C , Wang W . Knockdown of circ‐UQCRC2 ameliorated lipopolysaccharide‐induced injury in MRC‐5 cells by the miR‐326/PDCD4/NF‐κB pathway. Int Immunopharmacol. 2021;97:107633. 33895481 10.1016/j.intimp.2021.107633

[24] Aldakheel FM , Thomas PS , Bourke JE , Matheson MC , Dharmage SC , Lowe AJ . Relationships between adult asthma and oxidative stress markers and pH in exhaled breath condensate: a systematic review. Allergy. 2016;71(6):741–757. 26896172 10.1111/all.12865

[25] Mishra V , Banga J , Silveyra P . Oxidative stress and cellular pathways of asthma and inflammation: therapeutic strategies and pharmacological targets. Pharmacol Ther. 2018;181:169–182. 28842273 10.1016/j.pharmthera.2017.08.011 PMC5743757

[26] de Groot LES , Sabogal Piñeros YS , Bal SM , van de Pol MA , Hamann J , Sterk PJ , et al. Do eosinophils contribute to oxidative stress in mild asthma? Clin Exp Allergy. 2019;49(6):929–931. 30891863 10.1111/cea.13389 PMC6850153

[27] Fatani SH . Biomarkers of oxidative stress in acute and chronic bronchial asthma. J Asthma. 2014;51(6):578–584. 24593289 10.3109/02770903.2014.892965

[28] Jinhui S , Chunli C , Xiuxuan Y , Chunlong M , Jingwei W . STOML2 suppresses the proliferation, migration, and inflammation of airway smooth muscle cells in children with asthma by inhibiting NLRP3. J Biol Regul Homeost Agents. 2023;37(12):6871–6880.

[29] Yao L , Wang S , Wei P , Bao K , Yuan W , Wang X , et al. Huangqi–Fangfeng protects against allergic airway remodeling through inhibiting epithelial‐mesenchymal transition process in mice via regulating epithelial derived TGF‐β1. Phytomedicine. 2019;64:153076. 31473579 10.1016/j.phymed.2019.153076

[30] Türkeli A , Yilmaz Ö , Karaman M , Kanik ET , Firinci F , İnan S , et al. Anti‐VEGF treatment suppresses remodeling factors and restores epithelial barrier function through the E‐cadherin/β‐catenin signaling axis in experimental asthma models. Exp Ther Med. 2021;22(1):689. 33986854 10.3892/etm.2021.10121 PMC8112133

[31] Pei D , Shu X , Gassama‐Diagne A , Thiery JP . Mesenchymal–epithelial transition in development and reprogramming. Nat Cell Biol. 2019;21(1):44–53. 30602762 10.1038/s41556-018-0195-z

[32] Riemma MA , Cerqua I , Romano B , Irollo E , Bertolino A , Camerlingo R , et al. Sphingosine‐1‐phosphate/TGF‐β axis drives epithelial mesenchymal transition in asthma‐like disease. Br J Pharmacol. 2022;179(8):1753–1768. 34825370 10.1111/bph.15754 PMC9306821

[33] Tay Y , Rinn J , Pandolfi PP . The multilayered complexity of ceRNA crosstalk and competition. Nature. 2014;505(7483):344–352. 24429633 10.1038/nature12986 PMC4113481

[34] Xu X , Wang H , Liu S , Xing C , Liu Y , Aodengqimuge , et al. TP53‐dependent autophagy links the ATR‐CHEK1 axis activation to proinflammatory VEGFA production in human bronchial epithelial cells exposed to fine particulate matter (PM2.5). Autophagy. 2016;12(10):1832–1848. 27463284 10.1080/15548627.2016.1204496 PMC5079665

[35] Lee CG , Ma B , Takyar S , Ahangari F , Delacruz C , He CH , et al. Studies of vascular endothelial growth factor in asthma and chronic obstructive pulmonary disease. Proc Am Thorac Soc. 2011;8(6):512–515. 22052929 10.1513/pats.201102-018MW PMC3359071

[36] Haigh JJ . Role of VEGF in organogenesis. Organogenesis. 2008;4(4):247–256. 19337405 10.4161/org.4.4.7415 PMC2634330

[37] Edwards MR , Bartlett NW , Clarke D , Birrell M , Belvisi M , Johnston SL . Targeting the NF‐κB pathway in asthma and chronic obstructive pulmonary disease. Pharmacol Ther. 2009;121(1):1–13. 18950657 10.1016/j.pharmthera.2008.09.003 PMC7172981

